# The role of alpha-7 nicotinic receptors in food intake behaviors

**DOI:** 10.3389/fpsyg.2014.00553

**Published:** 2014-06-06

**Authors:** Kristina L. McFadden, Marc-Andre Cornier, Jason R. Tregellas

**Affiliations:** ^1^Department of Psychiatry, School of Medicine, University of Colorado – Anschutz Medical CampusAurora, CO, USA; ^2^Division of Endocrinology, Metabolism and Diabetes, Department of Medicine, School of Medicine, University of Colorado – Anschutz Medical CampusAurora, CO, USA; ^3^Research Service, Veterans Affairs Medical CenterDenver, CO, USA

**Keywords:** α7 nicotinic receptor, nicotine, obesity, eating behaviors, food intake

## Abstract

Nicotine alters appetite and energy expenditure, leading to changes in body weight. While the exact mechanisms underlying these effects are not fully established, both central and peripheral involvement of the alpha-7 nicotinic acetylcholine receptor (α7nAChR) has been suggested. Centrally, the α7nAChR modulates activity of hypothalamic neurons involved in food intake regulation, including proopiomelanocortin and neuropeptide Y. α7nAChRs also modulate glutamatergic and dopaminergic systems controlling reward processes that affect food intake. Additionally, α7nAChRs are important peripheral mediators of chronic inflammation, a key contributor to health problems in obesity. This review focuses on nicotinic cholinergic effects on eating behaviors, specifically those involving the α7nAChR, with the hypothesis that α7nAChR agonism leads to appetite suppression. Recent studies are highlighted that identify links between α7nAChR expression and obesity, insulin resistance, and diabetes and describe early findings showing an α7nAChR agonist to be associated with reduced weight gain in a mouse model of diabetes. Given these effects, the α7nAChR may be a useful therapeutic target for strategies to treat and manage obesity.

## INTRODUCTION

Nicotine has long been known to affect energy balance and weight. Smokers, for example, weigh less than age- and sex-matched non-smokers (Albanes et al., 1987), while smoking cessation is associated with increased food intake and weight gain (Stamford et al., 1986; Williamson et al., 1991; Filozof et al., 2004). Given the strong link between smoking and reduced weight, many report using smoking for weight control, or avoid cessation due to fear of weight gain (Camp et al., 1993; Wiseman et al., 1998; Fulkerson and French, 2003). Experimentally, nicotine has been shown to suppress appetite, increase energy expenditure, and alter feeding patterns, which can lead to weight loss (Jo et al., 2002; Zoli and Picciotto, 2012). Despite these known effects, however, the mechanisms underlying nicotine’s effects on eating behaviors and obesity remain unclear. Nicotine acts on both high-affinity nicotinic cholinergic receptors, such as the α4-β2 receptor, and low-affinity receptors, such as the α7 receptor, both centrally and peripherally. Recent studies suggest that the alpha-7 nicotinic acetylcholine receptor (α7nAChR) may play a particularly prominent role in nicotinic effects on eating behaviors. As such, this review focuses on neuronal effects of nicotinic agents, especially those involving the α7nAChR, how stimulation of this receptor influences eating behaviors and weight, and the potential utility of α7nAChR agonists as a novel treatment strategy for obesity.

## ALPHA-7 NICOTINIC ACETYLCHOLINE RECEPTORS

Neuronal nicotinic acetylcholine receptors consist of ligand-gated ion channels that are activated by acetylcholine, but also respond to nicotine and similar compounds. These receptors are comprised of five transmembrane subunits arranged around a central pore (Paterson and Nordberg, 2000; Dani and Bertrand, 2007). These subunits include αβ combinations (α2–α6 and β2–β4), homomeric nAChRs (α7–α9), and a heteromer α combination (α9 with α10) (McGehee et al., 1995; Jones et al., 1999; Dani and Bertrand, 2007). The two main types of nAChRs found in the brain are α4–β2 receptors and α7 receptors (Jensen et al., 2005; Changeux, 2010). While different nAChR subtypes may affect circuits involved in feeding behavior (Jo et al., 2002; Mineur et al., 2011a,b; Zoli and Picciotto, 2012), this review will focus on α7nAChRs, which are receiving increased research attention for their involvement in eating behaviors and food intake.

## CENTRAL EFFECTS OF α7nAChRs ON EATING BEHAVIORS

Previous reviews have described peripheral effects of nicotine and other α7nAChR agonists on obesity and eating behaviors (Bencherif et al., 2011; Lakhan and Kirchgessner, 2011). As such, while recent evidence for peripheral effects will be briefly examined, the primary focus of this review will be on central effects. Overall, nicotine and other α7nAChR agonists appear to suppress appetite through numerous complex, interacting central pathways, particularly those in the hypothalamus, which plays a fundamental role in energy balance. When various interactions are jointly considered, activation of hypothalamic α7nAChRs is thought to result in overall increased inhibition of appetite circuits, resulting in decreased food intake (Jo et al., 2002). Stimulation of α7nAChRs may also reduce food intake via effects on reward pathways or cortical networks involved in eating behaviors.

## α7nAChR EFFECTS ON HYPOTHALAMIC NEUROPEPTIDES

Hypothalamic nuclei most associated with energy balance and feeding regulation include the lateral hypothalamus (LH), ventromedial hypothalamus (VMH), arcuate nucleus (ARC), and paraventricular nucleus (PVN). The LH is often simplistically described as the “hunger center” and the VMH the “satiety center” (Schwartz et al., 2000; Zoli and Picciotto, 2012). The ARC is a primary center for peripheral feeding signal integration (e.g., leptin, insulin) and contains neurons that stimulate feeding and those that inhibit feeding when activated, with projections to the PVN and LH (Schwartz et al., 2000; Kageyama et al., 2012; Zoli and Picciotto, 2012).

**FIGURE 1:**
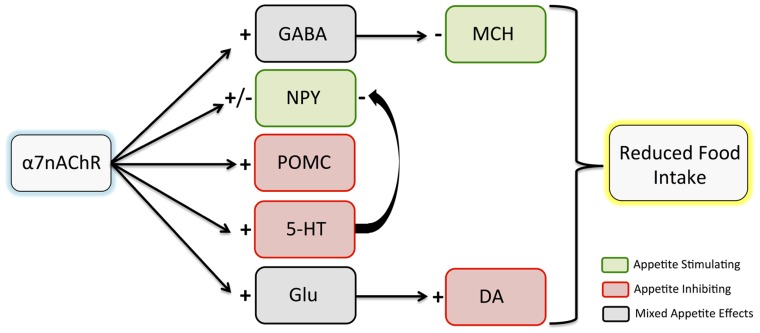
**Effects of α7nAChR stimulation on hypothalamic neuropeptide and neurotransmitter release.** Although there are complex interactions among pathways, it is hypothesized that the net effect of α7nAChR stimulation leads to reduced food intake. Inhibitory effects are indicated with a minus sign, while excitatory effects are indicated with a plus sign. Note that both inhibitory and excitatory effects of α7nAChR stimulation have been observed on NPY. While appetite effects of dopamine elsewhere in the brain are mixed, studies suggest that hypothalamic dopamine release contributes to appetite inhibition. α7nAChR, α7 nicotinic acetylcholine receptor; GABA, gamma aminobutyric acid; NPY, neuropeptide Y; POMC, proopiomelanocortin; 5-HT, serotonin; Glu, glutamate; MCH, melanin-concentrating hormone; DA, dopamine.

A primary potential pathway for α7nAChR mediation of eating behaviors involves hypothalamic cholinergic input. The hypothalamus contains rich cholinergic innervation and some of the highest levels of α7nAChR expression in the brain (Sargent, 1993). Appetite-related circuits within the hypothalamus can be modulated by nAChR activation, with a complex network of hormone and neuropeptide signals exerting neuronal effects to regulate eating behaviors. A number of studies have demonstrated effects of nicotine on these signals. Here, we will discuss α7nAChR involvement in cholinergic effects on proopiomelanocortin (POMC), neuropeptide Y (NPY), and melanin-concentrating hormone (MCH), all of which are involved in feeding regulation (**Figure 1**).

### POMC AND NPY

Nicotine may suppress appetite via activation of POMC neurons. POMC is produced in the hypothalamus (Huang et al., 2011; Zoli and Picciotto, 2012) and is a precursor for melanocortins, such as α-melanocyte-stimulating hormone (α-MSH), associated with suppressed food intake (Schwartz et al., 2000; Zoli and Picciotto, 2012). Electrophysiologically, Huang et al. (2011) demonstrated that nicotine excites mouse hypothalamic POMC neurons and that α7nAChRs are present on these neurons. Nicotine effects were reduced by the α7nAChR antagonist methyllycaconitine (MLA), suggesting at least partial mediation by α7nAChRs. As such, POMC stimulation is a potential mechanism through which α7nAChR agonism may suppress appetite. It should be noted, however, that MLA is not as selective an antagonist for α7nAChRs as α-bungarotoxin (Klink et al., 2001; Mogg et al., 2002), which should be considered when MLA is used to assess α7nAChR effects.

Neuropeptide Y, also produced in the hypothalamus, is associated with increased food intake (Schwartz et al., 2000). NPY neurons in the ARC project to the PVN to stimulate feeding (Morris, 1989; Kageyama et al., 2012). Thus, POMC and NPY have opposing effects on food intake. Smokers show reduced NPY levels compared to non-smokers, and smoking cessation is associated with increased NPY (Hussain et al., 2012), suggesting NPY inhibition as a mechanism for appetite suppression. However, nicotine effects on NPY are complex. As with POMC, NPY neurons in the hypothalamus are stimulated by nicotine and express α7nAChRs. Excitation of NPY neurons by nicotine is partially mediated by α7nAChRs, as MLA reduces excitation. Although nicotine reduces hypothalamic NPY mRNA in rats acutely (Frankish et al., 1995), NPY mRNA *increases* with chronic administration (Frankish et al., 1995; Li et al., 2000), which is accompanied by *decreased* food intake (Li et al., 2000). This is counterintuitive, as NPY stimulates food intake. However, nicotine also reduces hypothalamic NPY receptor density (Kane et al., 2001), which could explain the decreased intake. Another explanation for the net appetite-inhibiting effect of nicotine is that the depolarizing effect of nicotine on POMC neurons (anorexigenic) is significantly greater than that on NPY neurons (orexigenic). Furthermore, in addition to NPY neuron excitation by nicotine, inhibition of excitatory synaptic activity (glutamate release) on NPY neurons was also observed, an effect not seen in POMC neurons (Huang et al., 2011). Thus, although nicotine can excite NPY neurons, the greater direct excitation of appetite-inhibiting POMC neurons compared to appetite-stimulating NPY neurons, in addition to indirect inhibition of NPY neurons (via reduced glutamate release) may contribute to the net effect of appetite inhibition by nicotine and other α7nAChR agonists.

### MELANIN-CONCENTRATING HORMONE

Melanin-concentrating hormone (MCH) neurons are primarily located in the LH (Zamir et al., 1986) and also stimulate food intake (Qu et al., 1996). MCH may have a particular role in reward-related aspects of food, as MCH neurons project to the nucleus accumbens (NAC) and the ventral tegmental area (VTA), brain areas involved in reward processes (Schilstrom et al., 1998; Jo et al., 2005). MCH knockout mice are excessively lean and demonstrate reduced food intake (Shimada et al., 1998; Marsh et al., 2002). α7nAChRs may mediate gamma aminobutyric acid (GABA)-related inhibition of MCH neurons in the LH, leading to this appetite suppression (Jo et al., 2005).

## α7nAChR MODULATION OF NEUROTRANSMITTERS INVOLVED IN FOOD INTAKE BEHAVIORS

In addition to hypothalamic neuropeptides, nicotine modulates effects of multiple other neurotransmitter systems in the brain. The following section describes the impact of nicotine on GABA, glutamate, dopamine (DA), and serotonin, focusing on how α7nAChRs may inhibit appetite by modulating these neurotransmitter systems.

### GAMMA AMINOBUTYRIC ACID

Release of GABA, the main inhibitory neurotransmitter in the brain, is influenced by nAChRs (McGehee et al., 1995; Jones et al., 1999). Nicotine effects on appetite reduction may be associated with decreased excitability of MCH neurons in the LH via increased GABAergic inhibitory tone. Jo et al. (2005) found nicotine administration to facilitate GABAergic transmission in adult mice, and prenatal nicotine exposure to enhance postnatal GABAergic transmission. Specific involvement of α7nAChRs was also demonstrated, as an α7nAChR-specific antagonist (α-bungarotoxin) blocked these effects. As such, activation of α7nAChRs on GABAergic terminals in the hypothalamus may contribute to the anorexigenic effects of nicotine.

### GLUTAMATE AND DOPAMINE

Glutamate is the main excitatory neurotransmitter in the brain and plays a role in rewarding effects of nicotine, as nicotine increases glutamate release in the VTA and NAC, brain regions central to reward mechanisms (McGehee et al., 1995; Reid et al., 2000; Schilstrom et al., 2000). High concentrations of α7nAChRs are observed in the VTA (Clarke and Pert, 1985; Dominguez del Toro et al., 1994; Schilstrom et al., 1998; Jones and Wonnacott, 2004) and are thought to mediate nicotine-associated glutamate release (McGehee et al., 1995; Schilstrom et al., 2000). α7nAChR-mediated glutamate release plays a large role in nicotine’s effects on DA, a neurotransmitter critical in the reinforcing effects of nicotine (Schilstrom et al., 1998; Fowler et al., 2008). α4-β2nAChRs are sufficient for these reinforcing effects (Besson et al., 2012), likely via direct effects on DA neurons (Wooltorton et al., 2003; Besson et al., 2012). However, stimulation of α7nAChRs activates DA neurons via glutamatergic inputs (Yoshida et al., 1992; Schilstrom et al., 2000, 2003; Garzon et al., 2013). Thus, α7nAChR activation ultimately increases DA, but this is largely mediated via glutamatergic effects. Additionally, α7nAChRs may be important in dopaminergic function following long-term nicotine exposure, as they are more resistant to desensitization at usual levels for smokers than nAChR subunits containing β2 receptors, and may prevent dopaminergic hypoactivation resulting from chronic β2 desensitization (Besson et al., 2007, 2012).

The role of α7nAChR-mediated glutamate release in food consumption remains unclear. Administration of a glutamate *antagonist* has been found to increase food intake in rats (Maldonado-Irizarry et al., 1995; Stratford et al., 1998). As such, glutamate release stimulated by an α7nAChR *agonist* could decrease food intake. Increased DA release, amplified by α7nAChR-mediated glutamate release, increases the reward value of food (Yoshida et al., 1992; Schilstrom et al., 1998). Quarta et al. (2009) observed striatal DA release in mice following administration of an α7nAChR agonist (choline), an effect not observed in mice lacking α7nAChRs. Food-induced DA release is attenuated by an α7nAChR antagonist (MLA), implicating α7nAChRs in eating-related reward (Schilstrom et al., 1998). However, the role of DA in feeding behaviors is complex and varies by brain region. Although DA contributes to rewarding aspects of food intake in areas such as the VTA and NAC, hypothalamic DA release is though to contribute to nicotine-related reductions in food intake (Meguid et al., 2000; Schwartz et al., 2000). Thus, further study is needed to determine if effects of α7nAChRs on DA lead to overall increased or decreased consumption.

### SEROTONIN

Serotonin inhibits food intake (Waldbillig et al., 1981; Jo et al., 2002), likely by promoting satiety (i.e., meal stopping; Shor-Posner et al., 1986). One mechanism may be via NPY, as evidence suggests serotonin inhibits NPY release (Dryden et al., 1995, 1996a,b). Nicotine-induced nAChR activation can increase serotonin release, contributing to appetite suppression (Summers and Giacobini, 1995; Jo et al., 2002). Activation of α7nAChRs is thought to influence serotonin release, as α7nAChRs have been identified on serotonergic neurons (Galindo-Charles et al., 2008) and α7nAChR stimulation increases serotonin release in the dorsal raphe nucleus (Li et al., 1998).

## CORTICAL α7nAChR INVOLVEMENT IN FOOD INTAKE BEHAVIORS

Cortically, α7nAChR activation may affect limbic and paralimbic brain systems such as the insula and cingulate cortex, which also play a role in reward aspects of eating behaviors (Volkow et al., 2010) and contain rich cholinergic innervation (Nyback et al., 1989).

### INSULA/SALIENCE NETWORK

The insula, containing primary taste cortex, is involved in eating behavior regulation, including involvement in rewarding aspects of food and food-related arousal (Tataranni et al., 1999; Hinton et al., 2004; Cornier et al., 2009). The insula is also a central component of the salience network, an intrinsic brain network involved in assessing relevance of internal and external stimuli (Seeley et al., 2007; Bressler and Menon, 2010), in which altered response has been observed in obese, compared to lean, individuals (Garcia-Garcia et al., 2012; Kullmann et al., 2013). The insula is associated with urges and cravings related to both food and drugs of abuse (Pelchat et al., 2004; Naqvi and Bechara, 2009; Forget et al., 2010). Indeed, smokers sustaining insula damage following a stroke showed little subsequent difficulty quitting smoking, suggesting a role for the insula in effects of nicotine (Naqvi et al., 2007). However, the role of α7nAChRs in the insula is not yet known. Via α-bungarotoxin binding, studies have found α7nAChRs in the insula in both rats (Fuchs, 1989) and monkeys (Han et al., 2003). Presence of α7nAChRs in the human insula has been suggested by detection of α7nAChR mRNA (Wevers, 2011), but insular α7nAChR protein levels have not yet been studied in humans. As such, further study of α7nAChRs in the insula, and how activation of these receptors relates to eating behaviors, is needed.

### POSTERIOR CINGULATE/DEFAULT MODE NETWORK

The posterior cingulate cortex may also be involved in eating behaviors, having been associated with neuronal responses to visual food cues and taste (Tataranni et al., 1999; DelParigi et al., 2005; Cornier et al., 2009). The posterior cingulate is also a key component of the default mode network (DMN), an intrinsic brain network involved in self-referential thoughts and attention to internal stimuli (Buckner et al., 2008). DMN activity may play a role in eating behaviors, as overactivity of this network has been observed in obese, compared to lean, individuals (Tregellas et al., 2011a). Furthermore, this activity, which was associated with measures of appetite, was shown to change in response to feeding in lean, but not obese individuals. Nicotine can reduce resting-state DMN activity, including the posterior cingulate (Tanabe et al., 2011). α7nAChRs are present in high concentrations in the cingulate cortex, as assessed by α-bungarotoxin binding (Breese et al., 1997; Marutle et al., 2001). A study of DMN activity in schizophrenia patients observed reduced response following treatment with an α7nAChR partial agonist [3-2,4-dimethoxybenzylidene anabaseine (DMXB-A)], specifically in the posterior cingulate (Tregellas et al., 2011b). As with non-mentally ill obese individuals, DMN overactivity has been observed in schizophrenia patients (Garrity et al., 2007; Whitfield-Gabrieli et al., 2009), who are obese are rates twice those observed in the general population. Given these findings, it is possible that activation of α7nAChRs could be a mechanism to normalize DMN hyperactivity in obesity.

## α7nAChRs AND PERIPHERAL FACTORS INVOLVED IN EATING BEHAVIORS AND OBESITY

Recent studies have discovered a key role for α7nAChRs in peripheral factors related to obesity. In a mouse model of diabetes, Marrero et al. (2010) found that an α7nAChR-selective agonist (TC-7020) reduced weight gain and food intake, as well as glucose and triglyceride levels and expression of proinflammatory cytokines. These effects were reversed by an α7nAChR antagonist (MLA), supporting α7nAChR involvement. In humans, Cancello et al. (2012) have also found evidence supporting α7nAChR involvement in obesity. In addition to identifying α7nAChR expression in human mature adipocytes, they found that expression was downregulated in obese compared to lean adults, and that weight loss partially restored α7nAChR expression.

A potential mechanism through which peripheral α7nAChRs may exert weight and food intake effects is by mediating anti-inflammatory effects. Inflammation is a key feature of obesity, associated with increased proinflammatory cytokine production, insulin resistance, and development of type 2 diabetes (Marrero et al., 2010; Wang et al., 2011). Activation of α7nAChRs on cytokine-producing cells, such as macrophages, mediates this inflammatory response by inhibiting inflammatory cytokine production (Wang et al., 2011). A number of studies have demonstrated anti-inflammatory effects of nicotine (Wang et al., 2003; Lakhan and Kirchgessner, 2011) and smokers may have a reduced risk of some inflammatory diseases such as ulcerative colitis (Lakhan and Kirchgessner, 2011). The “cholinergic anti-inflammatory pathway” can be activated by α7nAChR agonists (Cheng et al., 2007). Supporting this, nicotine-induced cytokine inhibition can be blocked by α7nAChR-specific antagonists (Cheng et al., 2007), and α7nAChR knockout mice show increased LPS-induced proinflammatory cytokine production, including TNFα and IL-1β (Wang et al., 2003). Wang et al. (2011) found adipose tissue and macrophages in mice to express α7nAChRs, and while nicotine suppressed proinflammatory cytokine production, this effect was not observed in α7nAChR knockout mice. Additionally, nicotine reduced adipose tissue inflammation and improved insulin sensitivity in obese mice. Xu et al. (2012) observed improved insulin sensitivity in rodents following treatment with either nicotine or an α7nAChR agonist (PNU-282987), an effect not observed in α7nAChR knockout animals. These studies suggest that α7nAChRs are critical in anti-inflammatory effects of nicotine. Given this, therapeutics targeting α7nAChRs are increasingly being explored for diseases involving inflammation, such as diabetes, arthritis, and ulcerative colitis (Wang et al., 2003; Marrero et al., 2010; Bencherif et al., 2011; Lakhan and Kirchgessner, 2011).

## CONCLUSION

The α7nAChR plays an important role in both central and peripheral mechanisms involved in eating behaviors and energy balance. Studies have found links between α7nAChR expression and obesity, insulin resistance, and diabetes. Centrally, α7nAChRs modulate hypothalamic neuropeptides and neurotransmitters involved in feeding regulation and play a role in cortical processes affecting intake behavior. Overall, although the circuits involved are complex, it appears that net effects of nicotine and other α7nAChR agonists result in appetite suppression, which could lead to weight loss. Peripherally, and perhaps also centrally, α7nAChRs are also an important mediator of inflammation, a key contributor to health problems in obesity.

Although α7nAChR agonists have not yet been investigated for eating behavior effects in humans, preliminary animal work supports this idea, finding peripheral effects such as improved insulin sensitivity (Wang et al., 2011; Xu et al., 2012) and reduced weight gain and metabolic changes in a model of diabetes (Marrero et al., 2010). Further support for extending α7nAChR studies to humans lies in the observation that α7nAChRs are downregulated in human obesity, but normalize with weight loss (Cancello et al., 2012). In conclusion, given nicotine’s effects in humans, experimental support for α7nAChR involvement in eating behavior regulation, and early evidence of α7nAChR agonist effects in animal studies, the α7nAChR may represent a promising new therapeutic target for weight management and the treatment of obesity in humans.

## Conflict of Interest Statement

The authors declare that the research was conducted in the absence of any commercial or financial relationships that could be construed as a potential conflict of interest.
